# Primary Intrarenal Neuroblastoma in a Four-Month-Old Infant: A Rare Diagnostic Challenge Mimicking Wilms Tumor

**DOI:** 10.7759/cureus.81870

**Published:** 2025-04-08

**Authors:** Munir Ahmad, Mohammed Alblooshi, Abdalla Aboelkheir, Masih Abdul Kader

**Affiliations:** 1 Department of Pediatric Surgery and Urology, Al Jalila Children's Speciality Hospital, Dubai, ARE; 2 Department of Surgery, Tawam Hospital, Al Ain, ARE; 3 Department of Urology, Al Jalila Children's Speciality Hospital, Dubai, ARE

**Keywords:** catecholamine metabolites, intrarenal neuroblastoma, mycn status, pediatric renal tumor, wilms tumor mimic

## Abstract

Primary intrarenal neuroblastoma is an exceedingly rare entity that often mimics Wilms tumor in clinical and radiologic presentation, making prompt differentiation crucial given their divergent treatment pathways and prognostic implications. We present the case of a four-month-old male infant incidentally discovered to have a right-sided abdominal mass. Imaging suggested a renal malignancy, most likely Wilms tumor, but urgent surgical intervention was required due to intralesional bleeding and a precipitous drop in hemoglobin. Elevated urine catecholamine metabolites (homovanillic acid and vanillylmandelic acid) were subsequently detected, but only after the decision for surgery. Intraoperatively, the mass was confirmed to arise from the renal parenchyma rather than the adrenal gland. Pathologic examination revealed sheets of small round blue cells positive for chromogranin, synaptophysin, and cluster of differentiation 56, establishing the diagnosis of neuroblastoma. Four lymph nodes were positive for metastatic involvement, but there was no amplification of the MYCN oncogene. Postoperative urinary catecholamine metabolite levels normalized, and follow-up imaging demonstrated no residual disease at ten months. This case highlights the importance of considering primary intrarenal neuroblastoma in the differential diagnosis of pediatric renal masses and underscores the need for comprehensive imaging and laboratory evaluation to guide appropriate surgical management and postoperative surveillance.

## Introduction

Tumors presenting as renal masses in infants and young children pose a formidable diagnostic challenge given the broad differential diagnoses in this age group. Wilms tumor (nephroblastoma) is by far the most common malignant renal neoplasm in childhood, accounting for up to 90% of pediatric renal malignancies [1]. By contrast, primary intrarenal neuroblastoma is exceedingly rare, with published estimates suggesting it represents only 1-2% of all abdominal neuroblastomas [2]. Although neuroblastoma itself most frequently arises from the adrenal gland or sympathetic chain, occasional ectopic nests of primordial neural crest cells within the renal parenchyma can give rise to intrarenal neuroblastoma [3].

Clinically and radiologically, intrarenal neuroblastoma can be indistinguishable from Wilms tumor. Both entities may present as a flank mass in an otherwise healthy infant, often discovered incidentally or during routine evaluation for nonspecific abdominal symptoms. On ultrasound, Wilms tumor often appears as a well-defined intrarenal lesion, whereas neuroblastoma may show heterogeneous echotexture and, in extra-adrenal sites, can extend around vessels. Computed tomography (CT) and magnetic resonance imaging (MRI) can reveal calcifications more frequently in neuroblastoma and a propensity for encasement of vascular structures, but distinguishing an intrarenal neuroblastoma from Wilms tumor purely on imaging alone remains challenging [4]. Furthermore, hypertension (commonly associated with renal masses in children) is variably present in intrarenal neuroblastoma, adding another layer of diagnostic complexity [5]. While elevated urinary catecholamine metabolites (homovanillic acid and vanillylmandelic acid) are supportive of neuroblastoma, these can be normal in a subset of patients [6,7].

Timely and accurate distinction between Wilms tumor and intrarenal neuroblastoma is crucial because treatment paradigms and prognostic implications differ substantially. In Wilms tumor, neoadjuvant chemotherapy and nephron-sparing approaches are often considered when feasible, whereas definitive surgical resection or combined modality therapy remains central to managing high-risk neuroblastoma [1]. Herein, we report a case of primary intrarenal neuroblastoma in a four-month-old infant that clinically and radiologically mimicked Wilms tumor. Our aim is to highlight the diagnostic pitfalls of this uncommon entity and underscore the importance of maintaining a high index of suspicion when evaluating renal masses in very young children.

## Case presentation

A four‐month‐old male infant presented with a right‐sided abdominal mass that was discovered incidentally by his mother. He was born at term with a birth weight of 3.3 kg. Antenatal ultrasound scans were unremarkable, and there were no significant neonatal issues. On examination, the child appeared well, with no obvious distress or abnormal vital signs; however, a large, firm, and non‐tender mass was palpable in the right flank.

He initially visited a private clinic for a mild upper respiratory tract infection, where the flank mass was noted on routine examination. Despite the large mass, he remained mostly asymptomatic and continued to feed well on breast milk, with no vomiting, diarrhea, or constipation reported. Over the following days, however, he became less active and developed worsening anemia, as his hemoglobin dropped from 9.7 g/dL to 6.1 g/dL. On the day of admission, his abdomen was soft and non-tender, and his vital signs remained stable. This decline in hemoglobin, together with the mass’s apparent growth, prompted urgent inpatient evaluation and eventual surgical planning.

Initial laboratory workup revealed normal renal and liver function tests, and routine urinalysis was unremarkable. Hemoglobin was reduced, likely secondary to intratumoral hemorrhage, necessitating a packed red blood cell transfusion. Subsequent spot measurements of urinary homovanillic acid (HVA) and vanillylmandelic acid (VMA) showed elevated levels, raising suspicion of a neuroendocrine tumor origin.

Imaging findings

**Figure 1:**
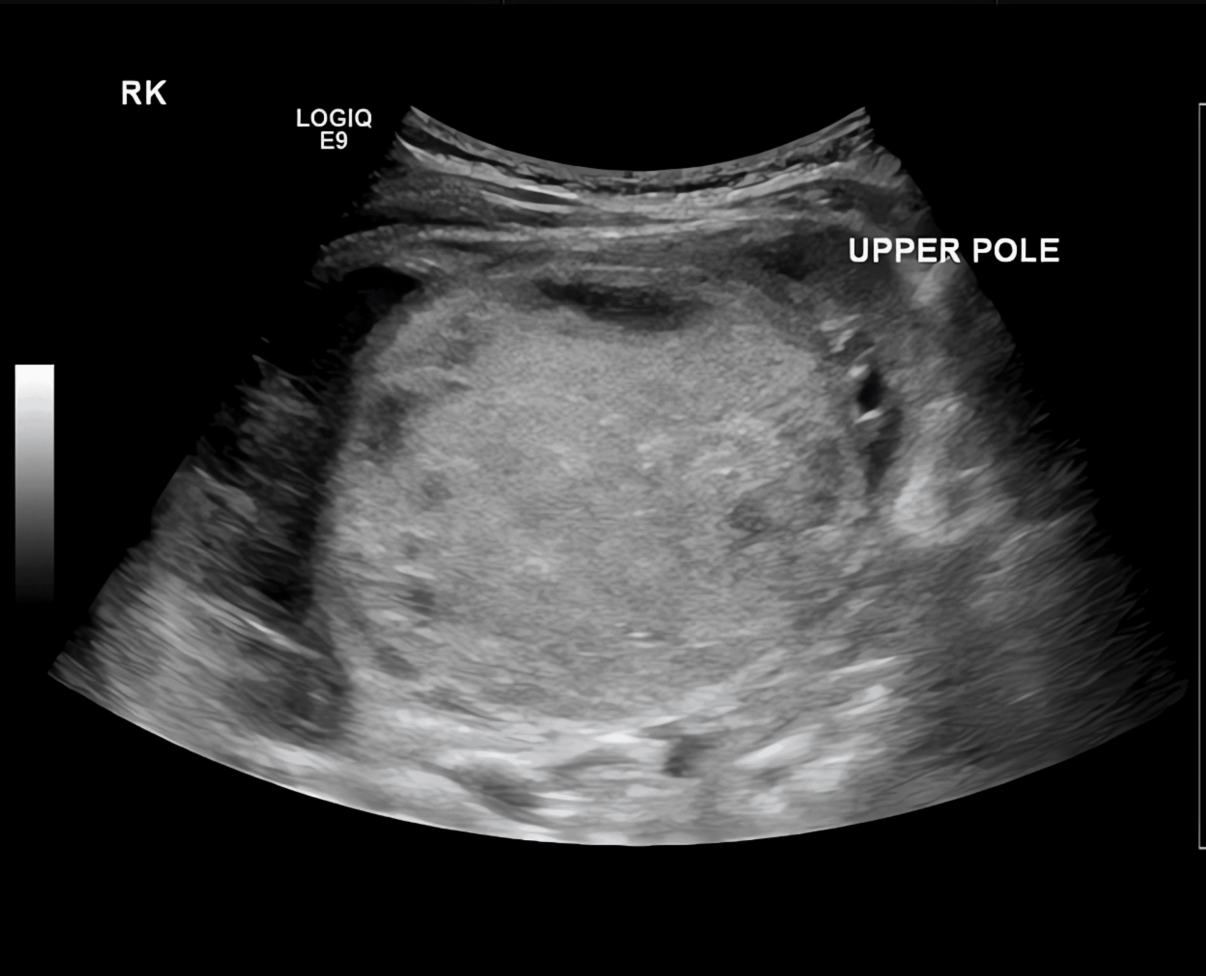
Ultrasound image of the right kidney showing a predominantly echogenic renal mass with small cystic/necrotic areas and mild hydronephrosis

Ultrasound (Figure 1) demonstrated a predominantly echogenic vascular mass in the lower pole of the right kidney, measuring approximately 5.9 × 4.7 cm. There were small areas of necrosis, and the mass displaced the remaining renal parenchyma superiorly and medially. Mild dilation of the pelvicalyceal system (renal pelvis anteroposterior diameter 7.6 mm) was noted, without hydroureter. A multi‐septated perinephric fluid collection measuring 5.0 × 3.9 × 4.8 cm was also identified. The right renal vein and inferior vena cava (IVC) appeared patent and free of tumor thrombus. The contralateral kidney was normal.

**Figure 2:**
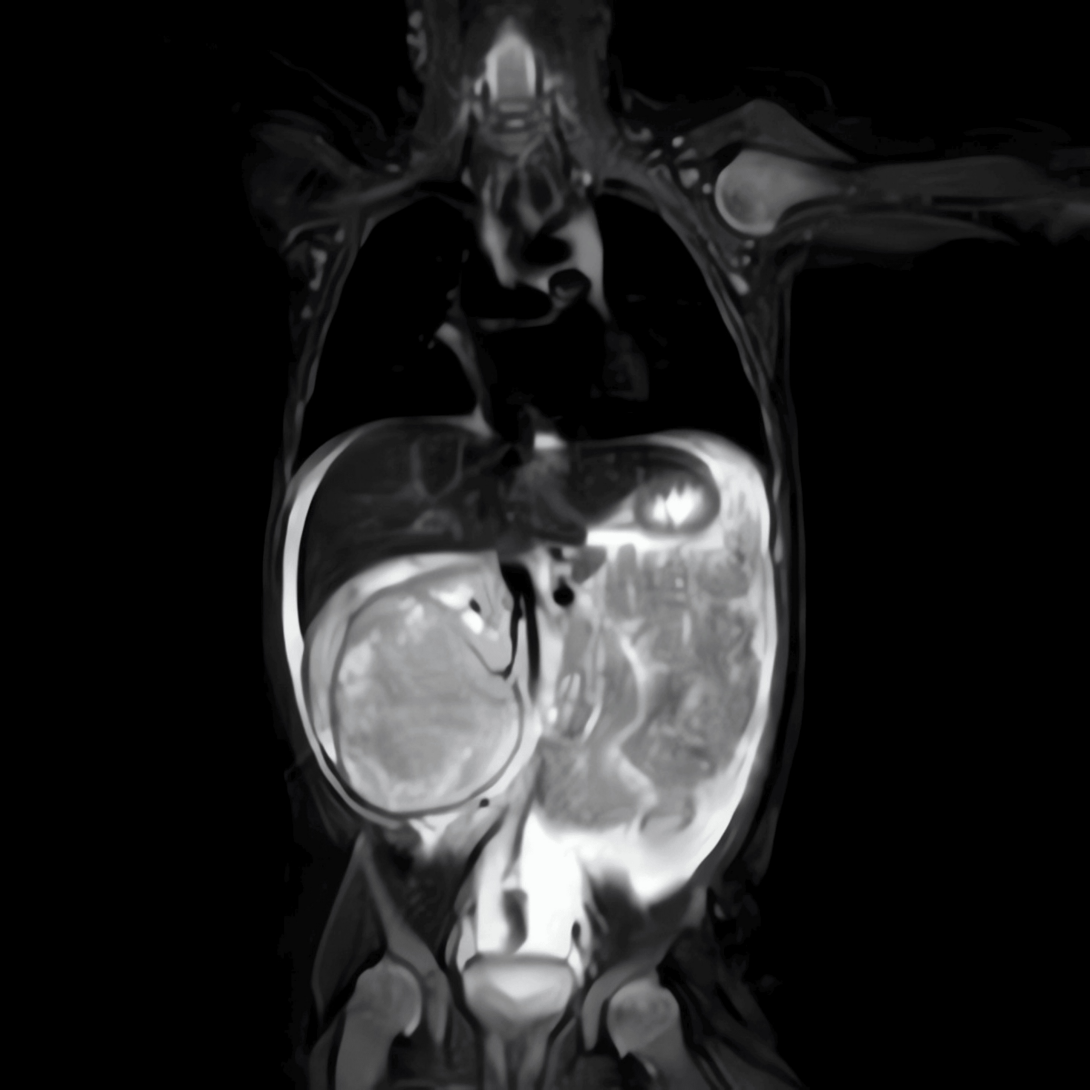
Coronal MRI of the abdomen demonstrating the large right renal tumor displacing surrounding structures

**Figure 3:**
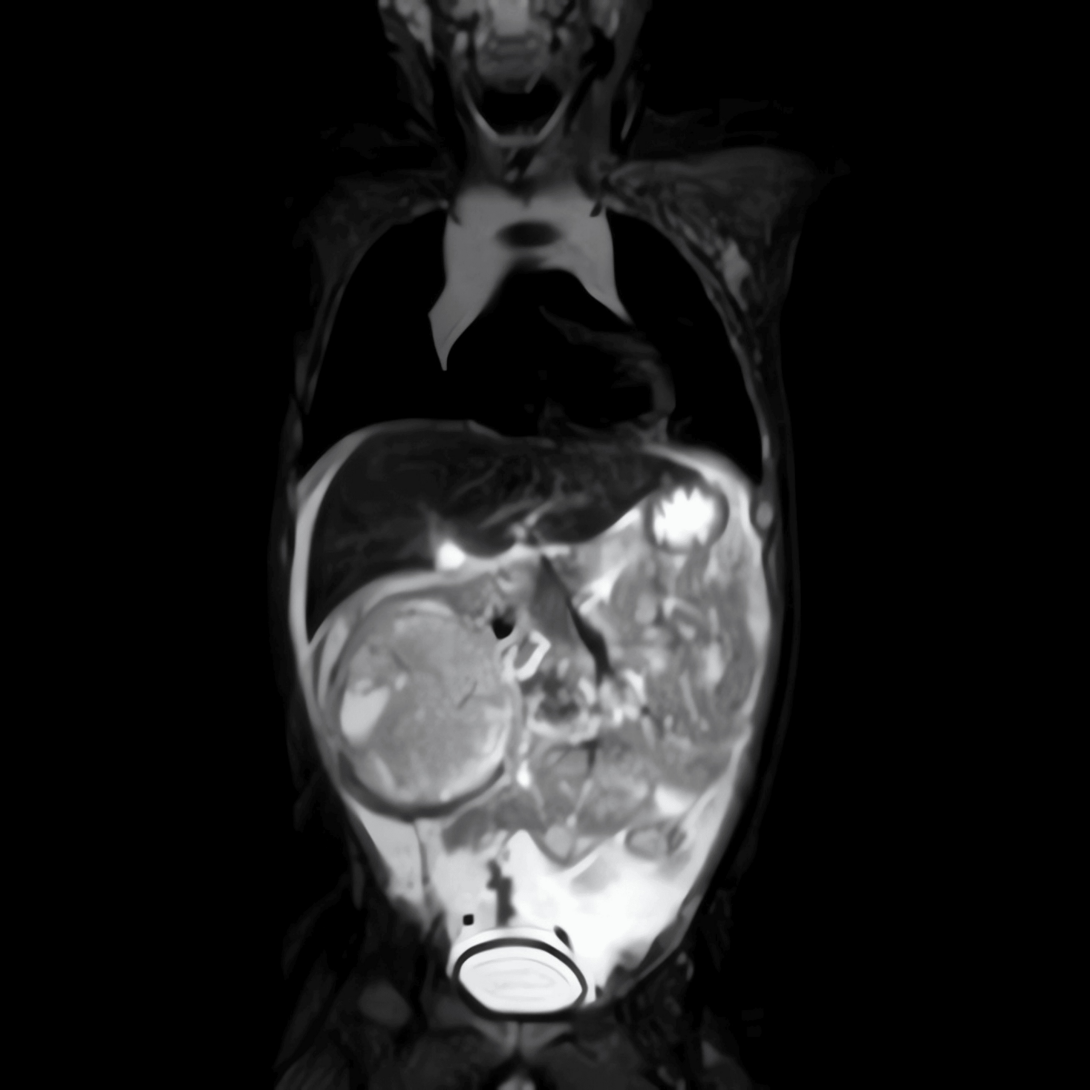
Coronal MRI of the abdomen with further characterization of the heterogeneous intrarenal mass and associated ascites

MRI of the abdomen and chest (Figures 2, 3) revealed an inhomogeneous neoplastic process within the right kidney, again measuring around 6 cm in maximum dimension. There was no extension into the renal vein or IVC. Moderate to large-volume ascites was present, although no metastatic disease was evident in the liver or lung fields.

Diagnostic decision-making

Despite the imaging findings indicating a likely renal malignancy, multiple diagnoses remained possible, including Wilms tumor and rare entities like intrarenal neuroblastoma. Ultrasound and MRI revealed a large lesion within the renal parenchyma, but these modalities alone could not distinguish definitively between Wilms tumor and neuroblastoma, as both can present with overlapping imaging features (e.g., heterogeneous intrarenal masses with areas of hemorrhage or necrosis). While calcifications or distinct vascular encasement patterns might point toward neuroblastoma, these were not clearly evident, adding to the diagnostic uncertainty. In addition, the child’s progressively declining hemoglobin due to suspected intratumoral bleeding introduced urgency. Further biopsies or more specialized imaging studies (e.g., MIBG scan) were deemed less practical given the risk of ongoing hemorrhage. Consequently, the medical team opted for an urgent surgical exploration and resection to both confirm the diagnosis and manage the potentially life-threatening anemia.

Operative findings

Because of progressive anemia attributed to tumor hemorrhage, an exploratory laparotomy was performed. We considered the risk of ongoing bleeding and a further drop in hemoglobin too high to wait for more tests. The child’s falling hematocrit and the large tumor size made urgent surgery necessary. Intraoperatively, the right adrenal gland appeared normal, separate from the tumor, which arose entirely from the renal parenchyma. A right radical nephrectomy was undertaken, along with systematic lymph node dissection. Surgical clips were placed to mark the hilar region and the upper renal pole for future imaging reference. The operative report details the gross appearance of the tumor and the surrounding anatomy. The child recovered well postoperatively and was discharged with plans for regular surveillance.

Pathological evaluation

**Figure 4:**
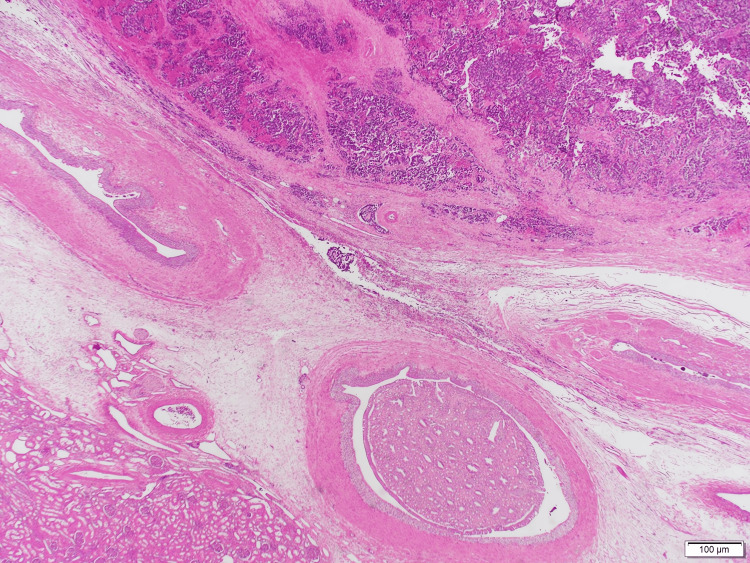
Low‐power photomicrograph showing normal renal parenchyma adjacent to sheets of small round blue cells (hematoxylin and eosin stain)

**Figure 5:**
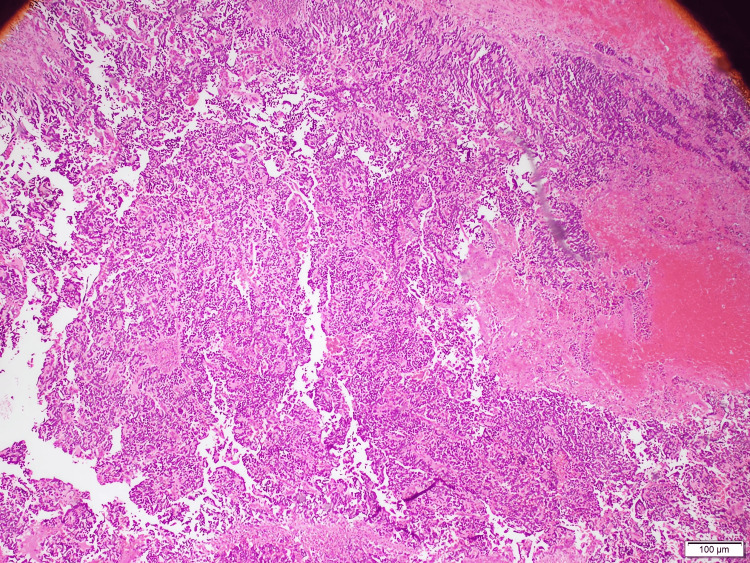
Low‐power view emphasizing the dense cellular architecture of the lesion, consistent with a small round blue cell tumor (hematoxylin and eosin stain)

**Figure 6:**
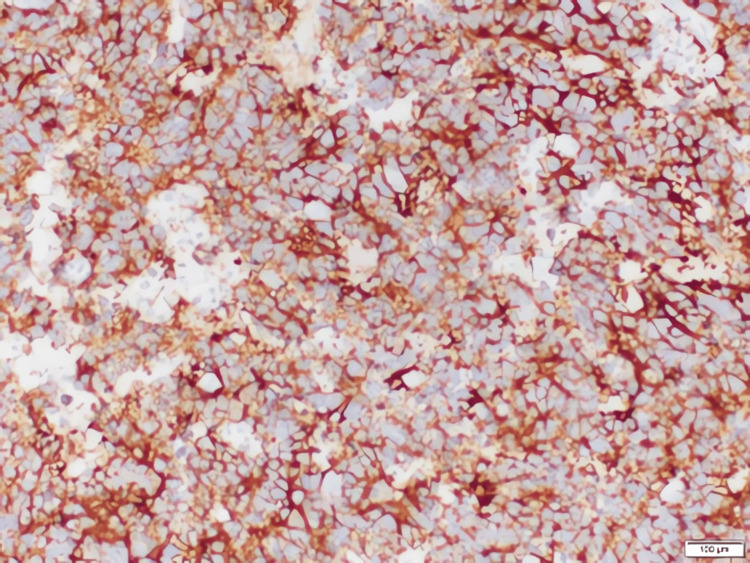
High‐power photomicrograph demonstrating diffuse chromogranin positivity within the tumor cells (immunohistochemical stain)

**Figure 7:**
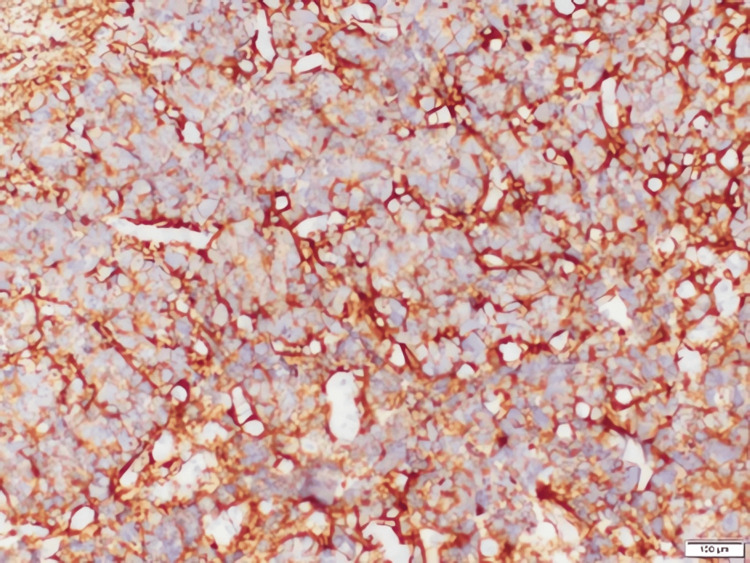
High‐power photomicrograph demonstrating synaptophysin positivity (immunohistochemical stain)

**Figure 8:**
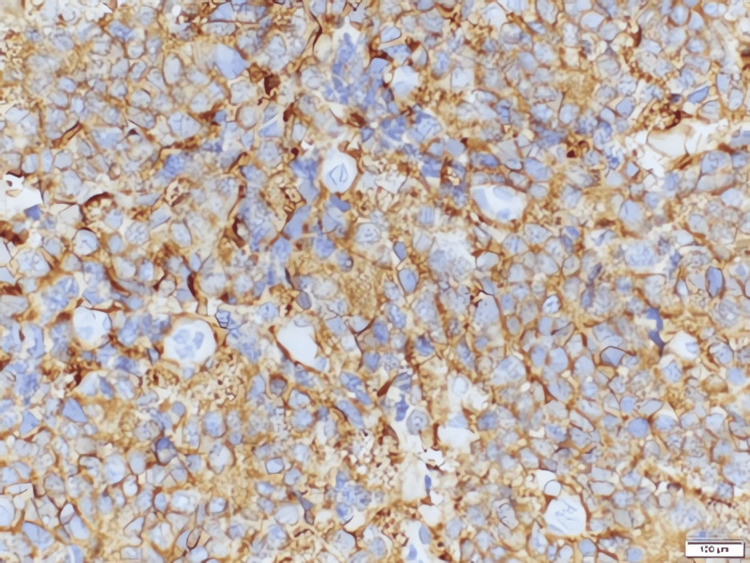
High‐power photomicrograph demonstrating robust CD56 immunostain positivity (immunohistochemical stain)

Grossly, the tumor was encapsulated but with areas of hemorrhage and necrosis. Microscopic examination showed residual, uninvolved renal parenchyma adjacent to sheets of small round blue cells (Figures 4, 5). Immunohistochemistry demonstrated strong positivity for neuroendocrine markers, including chromogranin (Figure 6), synaptophysin (Figure 7), and CD56 (Figure 8), confirming neuroblastoma. Additional immunostains such as neuron-specific enolase (NSE) and NB84 can further support a neuroblastoma diagnosis; however, these were not routinely performed in this case. Four of four hilar and paracaval lymph nodes were involved. Genetic testing (N‐myc fluorescence in situ hybridization) showed no evidence of N‐myc amplification, which is typically associated with aggressive disease. Other common neuroblastoma-associated genes (e.g., ALK, PHOX2B) were not evaluated in this instance, as MYCN amplification is the primary routine test at our institution.

Follow‐up

**Figure 9:**
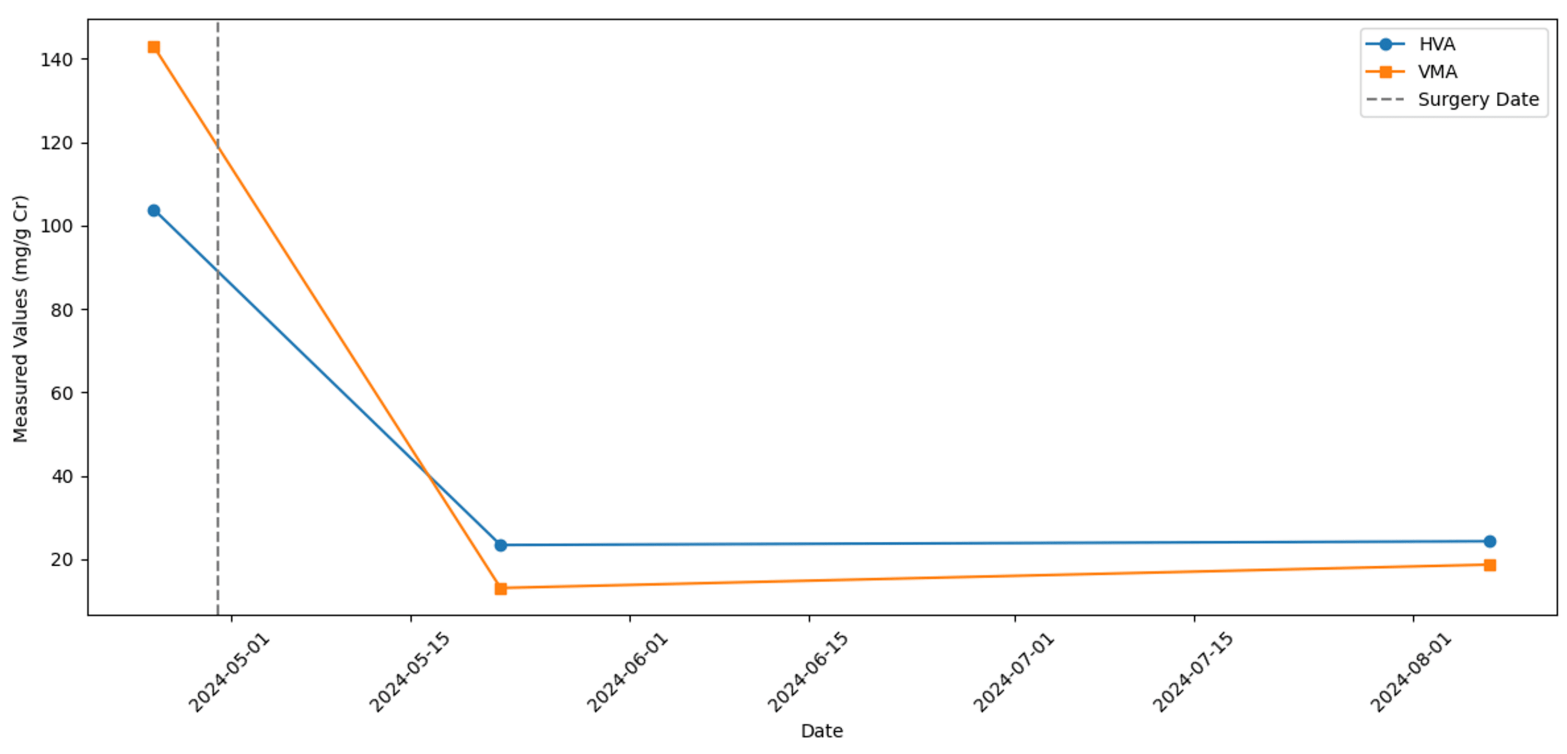
Graphical representation of urinary catecholamine metabolites (HVA and VMA) over time Line plots illustrating the urinary HVA (homovanillic acid) (red circles) and VMA (vanillylmandelic acid) (blue squares) levels at three time points: preoperatively (April 25, 2024), ~22 days postoperatively (May 22, 2024), and ~3 months postoperatively (August 7, 2024). The dashed vertical line marks the surgery date (April 30, 2024).

We present a timeline of homovanillic acid (HVA) and vanillylmandelic acid (VMA) values from initial diagnosis to post-operative follow-up (Figure 9). This visualization highlights the elevated preoperative levels (on April 25, 2024) and their normalization in subsequent measurements, underscoring the child’s favorable biochemical response following radical nephrectomy.

On April 25, 2024, the infant initially presented with markedly elevated urinary homovanillic acid (HVA, 103.9 mg/g Cr) and vanillylmandelic acid (VMA, 143 mg/g Cr). Owing to persistent intratumoral bleeding, a right radical nephrectomy was performed on April 30, 2024. Twenty-two days postoperatively, repeat urine testing demonstrated substantially reduced levels (HVA = 23.4 mg/g Cr, VMA = 13.1 mg/g Cr), and a Ga-68 DOTATOC PET-CT scan showed no evidence of active disease. Given his low‐intermediate risk profile (absence of MYCN amplification, favorable clinical response, and young age), the infant was placed on serial imaging and biochemical surveillance at three‐month intervals. At three months post‐surgery (August 7, 2024), urine HVA (24.3 mg/g Cr) and VMA (18.7 mg/g Cr) remained within normal limits, confirming a sustained biochemical remission. By the 10‐month follow‐up, there was no evidence of tumor recurrence or metastatic spread, further supporting a surveillance strategy with periodic imaging and urinary metabolite assessments.

## Discussion

Intrarenal neuroblastoma remains an exceedingly rare entity in pediatric oncology, with its presentation closely mimicking Wilms tumor (nephroblastoma) [4,7]. Clinically, both entities can manifest as a flank mass in an otherwise healthy infant or toddler, often discovered incidentally or during routine medical evaluations [2]. This overlapping presentation fosters a high degree of diagnostic complexity, necessitating careful integration of imaging, laboratory, and pathological data.

Although hypertension is frequently encountered in children with renal masses, especially those involving neuroendocrine origin, this sign may be absent, as was the case in our patient [5]. The utility of urinary catecholamine metabolites in differentiating neuroblastoma from other pediatric renal tumors is well recognized; however, these metabolites can be normal in a subset of neuroblastoma patients, particularly when sampling occurs at suboptimal times or when there is coexisting hemorrhage [6,7]. In our case, a precipitous drop in hemoglobin due to intralesional bleeding mandated urgent surgery, thereby limiting preoperative diagnostic workup. In practice, the measurement of urinary homovanillic acid and vanillylmandelic acid should be strongly considered for any child presenting with an atypical renal mass, given the profound therapeutic implications of identifying neuroblastoma.

Although neuroblastoma most commonly arises in the adrenal gland, primary intrarenal neuroblastoma is a rare subset that can be difficult to distinguish from Wilms tumor based on imaging alone. Wilms tumor (nephroblastoma) typically involves alterations in WT1 or WT2 genes (on chromosome 11) and often presents as an intrarenal, well-circumscribed mass with possible extension into the renal vein. By contrast, neuroblastoma frequently exhibits MYCN amplification (in aggressive cases) or alterations in ALK and PHOX2B, along with elevated urine catecholamine metabolites, which serve as a diagnostic clue. However, when neuroblastoma arises primarily within the renal parenchyma, classic radiologic features (e.g., calcifications and adrenal involvement) may be less apparent. From a clinical standpoint, children with Wilms tumor more commonly present with hematuria or hypertension, whereas neuroblastoma may be characterized by elevated urinary HVA and VMA. Treatment strategies differ: nephron-sparing approaches and chemotherapy often take precedence in Wilms tumor, while surgical resection plus risk-based chemotherapy and/or radiotherapy remain central for neuroblastoma.

Imaging tools such as ultrasound and MRI play a crucial role in the initial evaluation of pediatric renal masses. Whereas Wilms tumor can appear as a well-defined intrarenal lesion capable of displacing surrounding structures, neuroblastoma may show heterogeneous signal intensity, often with calcifications and a propensity to encase vital structures such as renal vessels [3,1]. However, when neuroblastoma arises primarily from renal tissue, these classic radiologic features can become ambiguous [4]. The lack of tumor thrombus in the renal vein or inferior vena cava in our patient initially supported the differential diagnosis of Wilms tumor, yet definitive preoperative distinction proved elusive until immunohistochemical staining confirmed a neuroendocrine phenotype.

From a surgical standpoint, radical nephrectomy is typically performed for large renal masses suspicious of malignancy. In this scenario, complete excision remains the cornerstone of curative management, whether the pathology reveals Wilms tumor or neuroblastoma [3]. However, opportunities for nephron-sparing approaches may arise in select cases, especially if neoadjuvant therapy reduces tumor burden significantly [1]. Future advances in molecular diagnostics, such as more rapid and accurate detection of tumor-specific genetic or metabolic markers, could allow for earlier recognition of intrarenal neuroblastoma, potentially altering treatment strategies to preserve renal function when feasible [8,9]. From a pathological standpoint, Wilms tumor often demonstrates immunoreactivity for WT1 in blastemal cells and may show expression of certain cytokeratins, whereas neuroblastoma cells consistently exhibit strong positivity for neuroendocrine markers (chromogranin, synaptophysin, CD56). This clear immunophenotypic contrast helped confirm the diagnosis of neuroblastoma in our patient, particularly given the small round blue cell morphology seen on microscopy.

Prognostically, primary intrarenal neuroblastoma may behave similarly to extra-adrenal neuroblastoma, with factors such as age, stage, histopathology, and the presence of genetic alterations (e.g., MYCN amplification) guiding risk stratification [1]. Our patient’s young age, absence of N-myc amplification, and normalization of urinary catecholamines postoperatively suggest a favorable long-term outlook. Nonetheless, diligent surveillance with imaging and biochemical tests remains essential to promptly detect any early relapse or metastatic disease.

This case underscores the diagnostic challenge posed by intrarenal neuroblastoma, particularly when presenting in a manner that mimics Wilms tumor [4,7]. Heightened clinical suspicion, complete radiologic assessment, and laboratory evaluation, including urinary catecholamine metabolite levels, are paramount for ensuring accurate diagnosis and appropriate treatment. Further research and accumulation of case series are crucial to refine best-practice guidelines for managing this rare tumor and improving outcomes in affected infants.

## Conclusions

Primary intrarenal neuroblastoma remains a rare yet significant diagnostic challenge that can easily be confused with Wilms tumor. Complete surgical excision, followed by tailored surveillance based on histopathological and genetic risk factors, is crucial to optimizing outcomes. This case highlights the need for a high index of suspicion when evaluating renal masses in infants, prompt and thorough diagnostic workup, including assessment of urinary catecholamine metabolites, and continued multidisciplinary follow‐up to detect and manage potential recurrence.
